# Obstinate Quartan Successfully Treated with Chloride of Sodium

**Published:** 1854-10

**Authors:** J. S. Wilson

**Affiliations:** Ala.


From the Southern Med. and Surg. Jour.

*Obstinate Quartan successfully treated with Chloride of Sodium.* By J. S. Wilson, M.D., of Ala.

Participating in the desire. now so prevalent in the profession, to obtain that great *desideratum*, a cheap and efficient substitute for the costly operations of Cinchona, in the treatment of intermittents, I have been induced to try common salt, in accordance with the recommendations of M. Piorry, Professor Dugas, and others. And as the desire alluded to can be consummated only by experiments and reports, to which each should contribute a share, I hope that no apology is necessary for reporting even a single case, especially when its interest is somewhat enhanced by an obstinacy which defiied the more ordinary and established remedies.

CASE.—On the 24th of March I was requested to prescribe for G. W., a young man of sanguine temperament, and of sound constitution naturally; but this had been impaired by frequent attacks of intermittent fever, which had produced, as usual, a pale cheek, and tumid spleen. These effects were accompanied by headache, mental and corporeal torpor, together with that indescribable sense of general indisposition characteristic of this abominable disease. And, as an evidence of the severity of the paroxysms, it may be added, that the last mentioned symptoms were persistent, continuing more or less on his "well days." He stated that several physicians had prescribed for him—that they had given him opium, and (perhaps) quinine, &c., &c., without "breaking them;" and I had myself, several weeks previously, put him on Fowler's solution, with the same unsuccessful result.

*Prescription:* Blue mass. 10 grs., to be followed by castor oil if necessary. Then, on chill-day, (26th,) begin ten hours before the time of the paroxysm, and take one of the following powders every two hours, with camphor mixture and laudanum, in willow-bark tea. ℞ Chloride of sodium, 360 grains. Make six powders.

27th. Says that he had his paroxysm at the usual time, and that it "shook him worse than usual;" pain in side (spleen) less, while fever was on: this was shorter also. These symptoms considered favorable.

*Prescription:* Omit all other remedies, and take 60 grs. chloride sodium, three times a day, on well days. Then begin nine hours before chill time, (29th,) and take 40 grs. of the same every hour, in warm gruel.

31st. Has had no paroxysm. Take Fowler's solution, 5 gtt. *ter in die*, as a prophylactic.

*Remark.*—The cure in this case must be attributed to the salt, as he took nothing else on the 29th; for it can hardly be supposed that the gruel had any agency in it.

